# Prevalence and risk factors for *Plasmodium falciparum* malaria in pregnant women attending antenatal clinic in Bobo-Dioulasso (Burkina Faso)

**DOI:** 10.1186/s12879-014-0631-z

**Published:** 2014-11-19

**Authors:** Mamoudou Cisse, Ibrahim Sangare, Guekoun Lougue, Sanata Bamba, Dramane Bayane, Robert Tinga Guiguemde

**Affiliations:** Centre MURAZ, Bobo-Dioulasso, Burkina Faso; Centre Hospitalier Universitaire Souro Sanou, Bobo-Dioulasso, Burkina Faso; Institut Supérieur des sciences de la Santé, Université Polytechnique de Bobo-Dioulasso, Bobo-Dioulasso, Burkina Faso; District sanitaire de Dafra, Bobo-Dioulasso, Burkina Faso

**Keywords:** Malaria, Prevalence, Pregnancy, Risk factors, Burkina Faso

## Abstract

**Background:**

Malaria during pregnancy remains a serious public health problem. The aim of this study was to determine the prevalence and possible risk factors for malaria in pregnant women attending antenatal clinic at two primary health facilities in Bobo-Dioulasso.

**Methods:**

We conducted a cross sectional study from September to December 2010 in two primary health facilities located in the periurban area of Bobo-Dioulasso. Pregnant women attending antenatal clinic (ANC) were included in the study after signing informed consent. For each participant, the social-demographic profile, malaria and obstetric histories were investigated through a questionnaire. Peripheral blood was collected and thick and thin blood smears were prepared to check *Plasmodium falciparum* parasitaemia. Hemoglobin concentration was measured. The associations between age, parity, gestational age, schooling, number of ANC visits, use of IPTp-SP, use of insecticide-treated nets (ITN) and anemia with the occurrence of *P. falciparum* malaria infection during pregnancy were analyzed through logistic regression.

**Results:**

During the period of study, 105 (18.1%) out of 579 pregnant women were infected by *P. falciparum*. The hemoglobin concentration mean was 10.5 ± 1.7/dL and was significantly lower in pregnant women with malaria infection (9.8 g/dL ±1.6) than in those who had no malaria infection (10.6 g/dL ±1.7) (P < 0.001). Multivariate analysis indicated that, education (AOR 1.9, 95% CI = [1.2-3.2]), parity [primigravidae (AOR 5.0, 95% CI = [2.5-9.8]) and secundigravidae (AOR 2.1, 95% CI = [1.2-3.8])], and anaemia (AOR 2.1, 95% CI = [1.3-3.5]) were significantly associated with *P. falciparum* malaria infection. The use of IPTp-SP was not associated with *P. falciparum* malaria infection.

**Conclusions:**

*P. falciparum* malaria infection is common in pregnant women attending antenatal clinic and anaemia is an important complication. The results show that the use of IPTp-SP does not reduce the risk of malaria incidence during pregnancy.

**Electronic supplementary material:**

The online version of this article (doi:10.1186/s12879-014-0631-z) contains supplementary material, which is available to authorized users.

## Background

Malaria during pregnancy is a serious public health problem in Sub-Saharan Africa. It is estimated that each year, approximately 25 million pregnant women in Sub-Saharan Africa are at risk of *Plasmodium falciparum* malaria infection during pregnancy [1]. Malaria during pregnancy leads to serious adverse effects on the mother and the child. Indeed although malaria during pregnancy might be asymptomatic due to high level of acquired immunity in mothers residing in high transmission areas, it is still associated with maternal anaemia, abortion, prematurity and low birth weight [1],[2]. Moreover, severe maternal anaemia increases the mother's risk of death and malaria related anaemia is estimated to cause as many as 10,000 maternal death each year in Africa [3]. In Burkina Faso, 22,130 cases of severe malaria were recorded during pregnancy in 2011 resulting in 97 maternal deaths [4].

To face this flail, the World Health Organization (WHO) adopted the intermittent preventive treatment with sulfadoxine-pyrimethamine during pregnancy (IPTp-SP) [3]. This policy has been adopted by the Burkina Faso ministry of health in 2005. Several epidemiological studies have been conducted in pregnancy before and after the implementation of IPTp-SP in Burkina Faso [5]-[17]. The prevalence of maternal peripheral *P. falciparum* infection assessed by microscopy was 24% in urban area [12] and varied from 19.4% to 50.8% in rural area [5]-[9],[13].

Factors influencing malaria prevalence in pregnant women include maternal age, parity, use of prophylaxis, nutrition, host and parasite genetics and level of anti-parasitic immunity [18] with conflicting data concerning maternal age and parity depending on urban or rural setting [19]-[23]. So far, only one study had investigated the risk factors of malaria during pregnancy in Burkina Faso [5]. This study had been carried out in a rural area and one year after the implemention of IPTp-SP pilot program.

Understanding the epidemiology of malaria during pregnancy facilitates decision on control strategies.This study aimed to assess the prevalence of *P. falciparum* malaria infection and possible associated risk factors in pregnant women attending antenatal clinic in the periurban area of Bobo-Dioulasso.

## Methods

### Study site and study design

A cross sectional study was performed from September to December 2010 in Lafiabougou and Kua two primary health facilities both located in the periurban area of Bobo-Dioulasso (Burkina Faso). In this area, the high transmission season of malaria lasts 4 months from August to November. The entomological inoculation rate (EIR) was about 63 infective bites per habitant per year "unpublished observations".

### Data collection

All pregnant women presenting for their routine antenatal clinic visits were included in the study after explaining to them the purpose of the study and signing informed consent. Each participant was evaluated only once. Data were obtained through a questionnaire and included the following information: social-demographic profile, malaria history, obstetric history including parity, gestational age. Gestational age was calculated from the first day of bleeding of the last menstrual period. Only two pregnant women refused to participate to the study because their husband did not allow them to participate to the study.

### Laboratory methods

Blood samples were taken by finger prick for checking both malaria parasite and hemoglobin concentration. Blood smear and thick drop assays were stained with 10% Giemsa dye. Smears were fixed with May Grunvald for three minutes before staining with Giemsa for 20 minutes. Parasite density was determined by counting asexual forms of the parasite per 200 leukocytes assuming 8,000 leukocytes/μL of blood. A slide was considered negative if no parasite was found after counting 500 leukocytes. All the slides were double-checked blindly and for discrepant results a third consensus reading was performed.

Hemoglobin concentration was determined by the hematic acid method using a hemoglobinometer (HemoCue AB, Angelhom, Sweden) and were classified as anemia (<11 g/dL), severe anemia (<8 g/dL) and normal (≥11 g/dL) [10].

### Sample size

The sample size calculation was based on an assumed prevalence of malaria in pregnant women attending antenatal clinic and benefiting from IPT-SP of 13.8% [5], a precision of 5% and for a significant result at the 5% level. A sample of 193 pregnant women was needed. Then we sampled 193 pregnant women in each trimester of pregnancy resulting a total size of 579 pregnant women.

### Statistical analysis of data

Data were double entered in EpiData 3.1 software and analyzed by using Stata 12 software.

The prevalence of *P. falciparum* malaria infection has been estimated with a 95% confidence interval (CI). Univariate analyses were performed by using the Pearson Chi-square or Fisher's exact tests to compare proportions for categorical variables. Comparison between continuous variables with normal distributions including age and hemoglobin concentration was done by the Student's t-test or the Anova test. The Wilcoxon rank sum test and the Kruskall-Wallis test were used to compare continuous variables with non-normal distributions (parasite density and number of ANC visits).

Simple and multiple logistic regression models were also used as described below. The variable malaria (including with and without *P. falciparum* malaria infection categories) was considered as the dependent variable. The independent variables included age, schooling, gestational age, parity, use of IPTp-SP and insecticide-treated net (ITN) during pregnancy, number of ANC visits and anaemia. Variables were categorized as follows: age (<20 years old, ≥20 years old); parity as primigravidae, secundigravidae, multigravidae (≥3); gestational age [first trimester (<14 weeks), second trimester (14-27 weeks) and third trimester (≥28 weeks)], number of ANC visits (0-1, 2 and ≥3). To investigate the association between the several independent variables and malaria, we began by performing simple logistic regressions with each independent variable. Next, we applied multiple regression models to control possible confusion. Variables exhibiting statistically significant associations (p < 0.05) or with important epidemiological meanings were included. These co-variables were kept in models, independent of their significance, in univariate analysis due to their possible relevance in the final results; thus, we could analyze their possible influence when considered together with the other variables.

### Ethical considerations

The study was initially discussed with health authorities and community leaders to obtain their assent. This study was approved by the National Ethics Committee for health research, Ouagadougou Burkina Faso (number 2010-054). A written informed consent was obtained from all pregnant prior to their enrolment in the study. For illiterate pregnant women, the informed consent discussion process was witnessed by an impartial individual. In those cases, the informed consent form had been signed with a thumbprint.

All women with anaemia or positive for malaria have been treated orally with folic acid plus ferrous and quinine 300 mg (24 mg/day until 7 days), respectively.

## Results

### Baseline characteristics of pregnant women attending antenatal clinic

The baseline characteristics of the 579 pregnant women involved in the study are summarized in Table 1. Briefly, the pregnant women were young (24.7 years ±5.7) and most of them had no formal education (61.1%), and were multigravidae (44.7%). Moreover 46.9% and 26.7% of pregnant women benefited from ITN and at least two doses of IPTp-SP, respectively. The proportion of pregnant women receiving the recommended 2 or 3 doses of IPTp-SP was higher during the 3rd trimester (p < 0.001). Of the 579 participating women, 50.4% reported attending ANC at least once during their pregnancy. The number of ANC visits varied from 0 to 2 with a median of 2 visits. Among ANC attendees, majority (39.9%) made their first visit during the second trimester. Only 2.8% of the ANC attendees had complete attendance (considered to be at least four ANC visits during pregnancy). The prevalence of *P. falciparum* parasitaemia was 18.1% (95% CI = [15.1-21.2]) and the geometric mean of the parasite density (GMPD) was 2254 parasites/μL (95% CI = [1755-2894]). The geometric mean of the density of *P. falciparum* parasitaemia was significantly lower in pregnant women who had used IPTp-SP (P <0.001). The haemoglobin concentration mean was significantly lower in pregnant women with malaria infection (9.8 g/dL ±1.6) than in those who had no malaria infection (10.6 g/dL ±1.7) (P <0.001).

**Table 1:** **Baseline characteristics of pregnant women attending ANC in Bobo-Dioulasso**

Characteristics	Total	1st	2nd	3rd	P
	(n = 579)	Trimester (n = 193)	Trimester (n = 193)	Trimester (n = 193)	
**Mean age (year),**	24.7 ± 5.7	24.5 ± 5.7	24.2 ± 5.4	25.4 ± 5.8	0.09^a^
**± SD**					
< 20	108 (18.6)	41 (21.2)	39 (20.2)	28 (14.5)	0.2^p^
≥ 20	471 (81.4)	152 (78.8)	154 (79.8)	165 (85.5)	
**Education**					0.5^p^
No formal schooling	353 (61.1)	113 (58.5)	124 (64.6)	116 (60.1)	
Formal schooling	225 (38.9)	80 (41.5)	68 (35.4)	77 (39.9)	
**Parity**					0.01^p^
Primigravidae	170 (29.4)	72 (37.3)	61 (31.6)	37 (19.1)	
Secundigravidae	150 (25.9)	43 (22.3)	48 (24.9)	59 (37.6)	
Multigravidae	259 (44.7)	78 (40.4)	84 (43.5)	97 (50.3)	
**ITN Use or ownership**	271 (46.9)	90 (46.9)	91 (47.2)	90 (46.6)	0.9^p^
**ANC visits**					
Median, quartiles	2 (0-2)	-	1 (1-2)	2 (1-3)	<0.001^w^
No prior ANC visit	287 (49.6)	190 (98.5)	88 (45.6)	9 (4.7)	
1	125 (21.6)	3 (1.5)	77 (39.9)	45 (23.3)	<0.001^f^
2	108 (18.6)	0 (0.0)	24 (12.4)	84 (43.5)	
≥ 3	59 (10.2)	0 (0.0)	4 (2.1)	55 (28.5)	
**Doses of IPTp-SP**					
0	352 (60.8)	193 (100.0)	139 (72.0)	20 (10.4)	<0.001^f^
1	124 (21.4)	0 (0.0)	46 (23.8)	78 (40.4)	
≥ 2	103 (17.8)	0 (0.0)	8 (4.2)	95 (49.2)	
**Peripheral parasitaemia**	105 (18.1)	46 (23.8)	37 (19.2)	22 (11.4)	0.006^p^
**GMPD/μL,**	2254	2300	1886	2913	0.3^k^
**[95% IC]**	[1755-2894]	[1600-3008]	[1253-2839]	[1475-5757]	
**Mean haemoglobin (g/dL), ± SD**	10.5 ± 1.7	11.0 ± 1.7	10.1 ± 1.6	10.3 ± 1.7	<0.001^a^
**Anaemia (% Hb <11 g/dL)**	346 (60.0)	90 (46.9)	132 (68.4)	124 (64.6)	<0.001^p^
**Moderate to severe anaemia (% Hb <8 g/dL)**	37 (6.4)	5 (2.6)	17 (8.8)	15 (7.8)	0.03^p^

^a^Determined by Anova Test. ^p^Determined by Pearson Chi-square Test.

^f^Comparison between 2nd and 3rd trimester determined by Chi-square (Fischer exact) Test.

^w^Determined by Wilcoxon rank sum Test. ^k^Determined by Kruskal-Wallis Test.

### IPTp-SP uptake at ANC clinic

The proportion of women receiving the recommended 2 or 3 doses of IPTp-SP increased (p < 0.001) with the number of ANC visits attended (Table 2).

**Table 2:** **Distribution of IPTp-SP doses according to the number of ANC visits**

Characteristics	Number of ANC visits			P
	0-1	2	≥ 3	
**IPTp-SP doses**				
0	351 (85.2)	1 (0.9)	0 (0.0)	<0.001^f^
1	59 (14.3)	53 (49.1)	12 (20.3)	
≥ 2	2 (0.5)	54 (50.0)	47 (79.7)	

^f^Determined by Chi-square (Fischer exact) Test.

### Factors associated with malaria among pregnant women attending antenatal clinic

Multivariate analysis indicated that, lower level of education (AOR 1.9, 95% CI = [1.2-3.2]), parity [primigravidae (AOR 5.0, 95% CI = [2.5-9.8]) and secundigravidae (AOR 2.1, 95% CI = [1.2-3.8])], and anaemia (AOR 2.1, 95% CI = [1.3-3.5]) were significantly associated with *P. falciparum* malaria infection. The use of IPTp-SP was not associated with *P. falciparum* malaria infection (Table 3).

**Table 3:** **Risk factors associated with malaria among pregnant women attending ANC in Bobo-Dioulasso**

Potential factors	Malaria	Crude OR	Adjusted OR	P
		[95% IC]	[95% IC]	
**Age**				
≥ 20 years	17.0 (80/471)	1	1	-
< 20 years	23.1 (25/108)	1.5 [0.9-2.4]*	1.6 [0.8-3.2]	0.2
**Education**				
Formal schooling	19.8 (70/353)	1	1	-
No formal schooling	15.6 (35/225)	1.3 [0.9-2.1]*	1.9 [1.2-3.2]	0.01
**Gestational age**				
3rd trimester	11.4 (22/193)	1	1	-
2nd trimester	19.7 (37/193)	1.8 [1.04-3.3]	0.7 [0.3-1.5]	0.4
1st trimester	23.8 (46/193)	2.4 [1.4-4.23]	0.8 [0.3-1.9]	0.6
**Parity**				
Multigravidae	11.2 (29/259)	1	1	1
Secundigravidae	18.0 (27/150)	1.7 [0.9-3.1]	2.1 [1.2-3.8]	0.02
Primigravidae	28.8 (49/170)	3.2 [1.9-5.3]	5.0 [2.5-9.8]	<0.001
**ANC visits**				
0-1	22.3 (92/412)	1	1	1
2	4.6 (5/108)	0.2 [0.1-0.4]	0.3 [0.1-1.0]	0.05
**≥** 3	13.6 (8/59)	0.5 [0.2-1.2]	1.3 [0.3-5.2]	0.7
**IPTp-SP dose**				
0	23.4 (83/352)	1	1	-
1	11.3 (14/124)	0.4 [0.2-0.8]	0.5 [0.2-1.2]	0.1
≥ 2	7.8 (8/103)	0.3 [0.1-0.6]	0.3 [0.1-1.5]	0.1
**ITN**				
No	20.9 (64/307)	1	1	1
Yes	14.8 (40/271)	0.7 [0.4-1.0]	0.8 [0.5-1.5]	0.4
**Anaemia**				
No	12.5 (29/231)	1	1	1
Yes	21.7 (76/346)	2.0 [1.2-3.1]	2.1 [1.3-3.5]	0.003

*P value =0.2.

## Discussion

This study aimed to assess the prevalence of *P. falciparum* malaria infection and possible associated risk factors in pregnant women attending ANC in the periurban area of Bobo-Dioulasso. The prevalence of maternal peripheral *P. falciparum* infection assessed by microscopy was 24% in urban area [12] and varied from 19.4% to 50.8% in rural area [5]-[9],[13]. An additional file shows this in more detail [see Additional file 1]. The higher observed rates in rural area could be explained by the high transmission level of malaria in rural area of Burkina Faso (unpublished observations). The difference between our results with those reported in Ouagadougou, the capital of Burkina Faso [12] could be due to the lower use of IPTp-SP in that study. Our rate was higher compared to that found in the rural area of Ghana in 2012 [24]. Indeed, all pregnant women included in the Ghanaian study had benefited from at least 2 doses IPTp-SP. A prevalence of 10.9% was found in Luanda, Angola, an urban site with similar malaria epidemiological characteristics [25] even though the survey had been conducted during high the transmission season of malaria. Our findings about the prevalence of malaria may be underestimated due to the use of microscopy of Giemsa-stained blood smears of peripheral blood for diagnosis. The use of PCR could have improved the sensitivity and specificity of the diagnosis as previously reported in Burkina Faso [13] nevertheless the high microscopic prevalence is of serious concern.

This study showed that lack of formal education was a risk factor for *P. falciparum* malaria.This agrees with previous findings in India [26] and highlights the need of more sensitization to increase the use of malaria prevention measures. Parity was associated with malaria infection even adjusted for age as previously shown [5]. Association between the decrease in risk of malaria infection and parity has been reported in several studies [27],[28]. However, in none of these studies, the association between parity and malaria infection was adjusted for women's age. A common explanation of the association between malaria infection and parity is that pregnancy is associated with a decrease in immunity [2]. In Mali, parity was significantly associated with malaria infection only when the analysis was not adjusted for age [21]. Other authors found no association between parity and malaria infection [19],[23],[25]. In the present study and as well as in that of Adam *et al*. [23], age was not significantly associated with malaria infection in contrast to the previous observations [19],[21],[22]. Previous sulfadoxine-pyrimethamine chemoprophylaxis was not associated with the prevalence of malaria infection when adjusted for different factors*.* In addition the coverage of the recommended 2 or 3 IPTp-SP among the 3rd trimester women was very low (49.2%) but it is higher than the recent reported coverage (10-19%) for Burkina Faso [29].

Furthermore only 46.6% of them reported ITN ownership. All these findings together highlight the WHO 2004 malaria prevention program failure and suggest the need to replace SP. However is it worth replacing SP when the WHO goal of four ANC visits [3] during pregnancy is not yet achieved? Indeed, the majority of the 3rd trimester women (71.5%) have attended less than three ANC visits. This is of serious concern because the cost of an ANC consultation is officially free of charge in Burkina Faso. The lower level of education of pregnant women could have contributed to the low level of correct ANC attendance. Thus educating pregnant women on focused ANC visits is recommended. Moreover the whole health system needs to be revitalized including antenatal care staffs that also need to be educated and enthused so that they can explain to women to try to meet the number of visits; the government needs to support access to antenatal care in rural areas.

The coverage of the recommended SP dosage increased with the number of ANC visits as previously reported in Benin [30] and in Cameroon [31]. However in Gabon, almost 40% of delivering women with more than three ANC visits had no or partial SP uptake [32].

This suggests that the number of ANC visits does not necessarily ensure the complete IPTp- SP coverage. Indeed, it has been shown that the coverage of IPTp-SP increases and ANC attendance is substantial when SP is supervised [32]. Thus further studies are needed to explore barriers to complete IPTp-SP coverage in Bobo-Dioulasso.

The mean haemoglobin was significantly lower in women with malaria infection. This agrees with findings not only in areas of stable transmission of malaria [25],[33] but also in areas with unstable transmission [23],[34]. Anaemia was the main complication of malaria infection found in our study and remains the most frequent consequence of malaria during pregnancy irrespective of transmission level and pre-pregnancy level of malaria immunity [35].

## Conclusion

Our results suggest that *P. falciparum* is common in pregnant women attending antenatal clinic in Bobo-Dioulasso and that anemia is an important complication associated with *P. falciparum* infection. Lack of formal education and parity even adjusted for age are main risk factors for malaria. The use of IPTp-SP was not associated with *P. falciparum* malaria infection.

## Additional file

## Electronic supplementary material

Additional file 1: **Prevalence of maternal peripheral*P. falciparum*infection from 2004 to 2011 in Burkina Faso.** The data provided show the prevalence of maternal peripheral *P. falciparum* infection reported in Burkina Faso, organized by type of study, period of study, location, pregnant women's data including age and parity. (PDF 13 KB)

## Abbreviations

ANC: Antenatal clinic
AOR: Adjusted odd ratio
CI: Confident interval
Dhfr: Dihydrofolate reductase
dL: Deciliter
g: Gramme
GMPD: Geometric mean of the parasite density
Hb: Hemoglobin
ITP-SP: Intermittent preventive treatment with sulfadoxine pyrimethamine
ITN: Insecticide-Treated Net
OR: Odd ratio
WHO: World Health Organization
