# Supplementary material for: Prevalence and risk factors for Plasmodium falciparum malaria in pregnant women attending antenatal clinic in Bobo-Dioulasso (Burkina Faso)
Source: BMC Infect Dis. 2014 Nov 19;14:631. doi: 10.1186/s12879-014-0631-z (PMC4240871; doi:10.1186/s12879-014-0631-z)
Supplement: Supplementary file 1 — Additional file 1: Prevalence of maternal peripheralP. falciparuminfection from 2004 to 2011 in Burkina Faso. The data provided show the prevalence of maternal peripheral P. falciparum infection reported in Burkina Faso, organized by type of study, period of study, location, pregnant women's data including age and parity. (PDF 13 KB) [file 12879_2014_631_MOESM1_ESM.pdf]

Table 1 : Prevalence of maternal peripheral *P. falciparum* infection from 2004 to 2011 in Burkina Faso

Prevalence of maternal peripheral *P. falciparum* infection	Type of study	Period of study	Location	Age	Parity	Reference
- 25.1 % (by microscopy) at ANC visit<br>- 19.4 % (by microscopy) at delivery	Cross sectional	June-November 2004	Rural	Median age : 25 years	All parity	Reviewed in [5]
- 23 % (by microscopy) at 32 weeks of gestation<br>- 23.2 % (by microscopy) at delivery	Clinical trial	2004-2006	Rural	Median age : 19 years	All parity	Reviewed in [6]
- 20 % (by microscopy) at 32 weeks of gestation<br>- 18 % (by microscopy) at delivery	Clinical trial	2004-2006	Rural	No data	Primigravidae and secundigravidae	Reviewed in [7]
- 50.8% (by microscopy) at enrolment (1st ANC visit)<br>- 21.9 % (by microscopy) at 2nd ANC visit	Clinical trial	April 2004 November 2005	Rural	Mean age : 23.7 ± 5.9 years	All parity	Reviewed in [8]
- 25.8 % (by microscopy) at delivery	Clinical trial	April 2004 November 2005	Rural	Mean age : 23.7 ± 5.9 years	All parity	Reviewed in [9]
- 24 % (by microscopy) at ANC visit<br>- 30 % (by HRP2 RDT) at ANC visit	Cross sectional	September-October 2010	Urban	Mean age : 25 ± 5 years	All parity	Reviewed in [12]
- 30 % (by microscopy) at ANC visit<br>- 47 % (by HRP2 RDT) at ANC visit<br>- 53 % (by RT-PCR) at ANC visit	Cross sectional	November 2010-August 2011	Rural	No data	All parity	Reviewed in [13]
